# Impact of beta blockers on cancer neuroimmunology: a systematic review and meta-analysis of survival outcomes and immune modulation

**DOI:** 10.3389/fimmu.2025.1635331

**Published:** 2025-08-06

**Authors:** Fangyuan Zhang, Yu Wang, Feng Liu, Yixian Li, Xinxin Liu, Xueying Ren, Xi Yuan

**Affiliations:** ^1^ Jiangsu Province Hospital of Chinese Medicine, Affiliated Hospital of Nanjing University of Chinese Medicine, Nanjing, China; ^2^ School of Pharmaceutical Science and Technology, Hangzhou Institute for Advanced Study, University of Chinese Academy of Sciences, Hangzhou, China; ^3^ Department of Oncology, Jinling Hospital, Affiliated Hospital of Medical School, Nanjing University, Nanjing, China; ^4^ Department of General Surgery, Affiliated Hospital of Nanjing University of Chinese Medicine, Nanjing, Jiangsu, China

**Keywords:** Beta-blockers, cancer neuroimmunology, immune checkpoint inhibitors, tumor microenvironment, meta-analysis

## Abstract

**Background:**

Emerging evidence suggests that beta-blockers (BBs) may influence cancer progression by modulating the neuroimmune axis. However, clinical findings remain heterogeneous, necessitating a comprehensive evaluation of their impact on survival outcomes and immune modulation across malignancies.

**Methods:**

We conducted a systematic review and meta-analysis following PRISMA guidelines, analyzing 79 studies from PubMed, Embase, and Web of Science. Pooled hazard ratios (HRs) for overall survival (OS) and cancer-specific survival (CSS) were calculated using random-effects models. Subgroup analyses explored effects by cancer type, BB class (non-selective vs. β1-selective), and concurrent immunotherapy. Immune biomarkers (e.g., PD-L1 expression, tumor-infiltrating lymphocytes) were qualitatively synthesized.

**Results:**

BB use showed no significant overall effect on CSS (HR = 0.97, 95% CI: 0.92–1.02) but exhibited substantial heterogeneity (I² = 80%). Protective associations were observed in breast cancer (HR = 0.27–0.50) and melanoma, while detrimental effects emerged in pancreatic and head/neck cancers (HR > 1.0). Clinically, BBs combined with immune checkpoint inhibitors (ICIs) improved survival (HR=0.91, 95% CI: 0.85–0.98), particularly in PD-L1+ tumors (OR=1.29 for enhanced expression). Non-selective BBs showed stronger immune modulation (CD8+ T-cell SMD=0.49 vs 0.22 for β1-selective).

**Conclusion:**

BBs demonstrate clinically meaningful benefits when combined with immunotherapy (HR=0.91) particularly in β2-AR+ melanoma and breast cancer, but show potential harm in pancreatic/head-neck cancers (HR>1.0). These results support preferential use of propranolol (20-40mg/day) in immunotherapy-treated melanoma, and avoidance of routine BB use in non-immunogenic tumors without adrenergic profiling. Prospective trials should validate these selection criteria.

## 1 Introduction

The emerging paradigm of cancer neuroimmunology has fundamentally transformed our understanding of tumor biology by revealing the profound influence of neural-immune interactions on cancer progression and therapeutic response. Within this conceptual framework, the sympathetic nervous system (SNS) and its neurotransmitters, particularly catecholamines, have been identified as critical mediators of tumor microenvironment (TME) dynamics (1). Adrenergic signaling, primarily through β-adrenergic receptors (β-ARs), orchestrates bidirectional communication between neural and immune cells, fostering an immunosuppressive niche that facilitates tumor immune evasion, angiogenesis, and metastatic dissemination (2).

Recent studies highlight that chronic stress and SNS overactivation exacerbate cancer progression by upregulating β-AR signaling, which in turn suppresses cytotoxic T-cell activity, promotes myeloid-derived suppressor cell (MDSC) accumulation, and enhances regulatory T-cell (Treg) infiltration (3). Mechanistically, β-adrenergic blockade has been shown to enhance dendritic cell maturation, restore CD8+ T cell effector function, reduce myeloid-derived suppressor cell accumulation, and decrease regulatory T cell infiltration within tumors. These immunomodulatory effects correlate with improved responses to immune checkpoint inhibitors in animal models, suggesting that β-blockers may function as biological response modifiers capable of overcoming resistance to cancer immunotherapy. The pleiotropic actions of β-blockers extend beyond direct immune effects to include inhibition of tumor-associated angiogenesis, reduction of matrix metalloproteinase activity, and suppression of epithelial-mesenchymal transition - all processes known to be influenced by neural signaling pathways (4, 5).

The clinical translation of these preclinical insights has yielded a complex and sometimes contradictory body of evidence regarding β-blockers’ anticancer efficacy (6). Observational studies in breast cancer have consistently shown an association between β-blocker use and reduced recurrence risk, improved metastasis-free survival, and enhanced responses to neoadjuvant chemotherapy (7). Similar benefits have been reported in melanoma, where β-blocker use correlates with improved outcomes in patients receiving immunotherapy. However, other malignancies such as pancreatic cancer and glioblastoma have shown less consistent patterns, with some studies even suggesting potential detrimental effects in certain contexts (8). This heterogeneity may reflect differences in tumor innervation patterns, adrenergic receptor expression profiles, baseline sympathetic tone, or the specific β-blocker compounds used. Additionally, the timing and duration of β-blocker exposure relative to cancer diagnosis and treatment may critically influence outcomes, as the neuroimmune landscape evolves throughout disease progression and therapeutic intervention (9).

The interaction between β-blockers and cancer immunotherapy represents a particularly compelling area of investigation within cancer neuroimmunology. Preclinical data suggest that β-adrenergic blockade can potentiate the effects of PD-1/PD-L1 inhibitors by reversing catecholamine-induced T cell exhaustion and reducing immunosuppressive cytokine production. Clinical studies have reported improved progression-free and overall survival in patients receiving combination therapy compared to immunotherapy alone, though these findings require validation in prospective trials (10). The mechanistic basis for this synergy appears rooted in the ability of β-blockers to mitigate multiple non-redundant immunosuppressive pathways simultaneously, creating a more permissive microenvironment for immune-mediated tumor control. This multimodal immunomodulation distinguishes β-blockers from more targeted immune therapies and positions them as potential broad-spectrum adjuvants to cancer immunotherapy (11, 12).

Critical gaps remain in our understanding of how β-blockers influence the neuroimmune axis across different cancer types and treatment contexts. While the protective effects of β-blockers are most established in highly innervated tumors such as breast and prostate cancers, the generalizability of these findings to less innervated malignancies remains uncertain. Similarly, the optimal timing, duration, and specific β-blocker regimen for maximal neuroimmune modulation have not been systematically investigated. The non-selective β-blocker propranolol has been the most studied in preclinical models due to its ability to block both β1 and β2 receptors, but comparative clinical data across different β-blocker classes are lacking. Furthermore, the potential for β-blockers to exert differential effects based on host factors such as chronic stress levels, genetic polymorphisms in adrenergic receptors, or baseline autonomic tone has not been adequately explored.

The current meta-analysis seeks to address these knowledge gaps by systematically evaluating the impact of β-blockers on survival outcomes across malignancies while specifically examining evidence for immune-mediated mechanisms. By aggregating data from diverse clinical and translational studies, we aim to determine whether the neuroimmunomodulatory effects observed in preclinical models translate into measurable clinical benefits. Particular attention will be paid to studies incorporating biomarkers of neuroimmune activity, such as tumor infiltrating lymphocytes, circulating catecholamines, or gene expression signatures related to adrenergic signaling (13). The analysis will also explore potential effect modifiers including cancer type, stage, β-blocker class and duration, and concurrent therapies. Through this comprehensive synthesis of existing evidence, we hope to clarify β-blockers’ role as modulators of cancer neuroimmunology and identify promising directions for future mechanistic and clinical investigation.

Cancer neuroimmunology represents a frontier in oncology that demands innovative approaches to therapeutic development and clinical trial design. Traditional paradigms that view the nervous and immune systems as separate entities must give way to more integrated models that acknowledge their constant cross-talk in health and disease. β-blockers, as one of the first pharmacologic tools to emerge from this conceptual shift, offer a unique opportunity to test the therapeutic potential of neuroimmune modulation. Their widespread availability, well-characterized safety profile, and low cost make them particularly attractive candidates for clinical translation. However, realizing this potential requires rigorous evidence synthesis to guide appropriate patient selection and treatment protocols. The current meta-analysis represents a critical step in this process, aiming to separate signal from noise in the complex relationship between adrenergic blockade, immune function, and cancer outcomes (5).

The biological rationale for β-blockers in cancer neuroimmunology extends beyond simple receptor antagonism to encompass broader effects on tumor-nerve interactions. Recent studies have revealed that tumors actively remodel their neural microenvironment through the secretion of neurotrophic factors, creating dense networks of adrenergic and sensory fibers that facilitate metastatic spread and therapeutic resistance (2, 8). β-blockers may disrupt this feed-forward cycle by inhibiting nerve sprouting and reducing neurotrophic factor production, thereby normalizing the neural-tumor interface. Simultaneously, their immunomodulatory effects help restore antitumor immunity in what becomes a dual-pronged attack on cancer progression. This multifaceted activity distinguishes β-blockers from more narrowly targeted therapies and may explain their apparent efficacy across diverse cancer types and treatment settings.

Methodological challenges abound in studying neuroimmune interventions like β-blockers, as traditional oncology trial designs often fail to capture the dynamic interplay between neural and immune systems. Most clinical studies to date have relied on incidental β-blocker use rather than prospective randomization, introducing potential confounding by indication. Additionally, few trials have incorporated comprehensive neuroimmune monitoring, making it difficult to establish causal relationships between adrenergic blockade, immune changes, and clinical outcomes. The current meta-analysis will employ advanced techniques to address these limitations, including individual patient data analysis where available and rigorous assessment of study quality. By applying neuroimmunological lenses to existing clinical data, we aim to extract new insights about β-blockers’ mechanisms of action and therapeutic potential (14).

As the field of cancer neuroimmunology advances, it becomes increasingly clear that neural regulation of immunity represents an underutilized therapeutic target in oncology. The sympathetic nervous system functions as a master regulator of immune function, with the capacity to suppress antitumor responses through multiple redundant pathways. β-blockers, by interrupting this neural control, may help “release the brakes” on antitumor immunity in a manner complementary to immune checkpoint inhibitors. Their effects on tumor vasculature, stromal remodeling, and metastatic niche formation further enhance their potential as multifunctional cancer therapeutics. The current systematic review will provide the most comprehensive evaluation to date of whether these promising preclinical observations translate into clinically meaningful benefits for cancer patients, while identifying key knowledge gaps that should guide future research in this emerging field.

## 2 Method

### 2.1 Meta-analysis guidelines

This meta-analysis was conducted following the Preferred Reporting Items for Systematic Reviews and Meta-Analyses (PRISMA) guidelines to ensure methodological rigor and transparency. A comprehensive search strategy was developed to identify all relevant studies evaluating the impact of β-blockers on survival outcomes and immune modulation in cancer patients.

### 2.2 Literature search strategy

Our search covered publications from database inception to March 1st, 2024, ensuring inclusion of all relevant studies published during the era of modern cancer immunotherapy. A systematic search was performed in PubMed/MEDLINE, Embase, Web of Science, and Cochrane Library from inception to [insert date]. The search strategy combined Medical Subject Headings (MeSH) terms and keywords related to β-blockers (e.g., “propranolol,” “beta-adrenergic blockade,” “atenolol”), cancer (e.g., “neoplasms,” “tumor,” “oncology”), and neuroimmunology (e.g., “neuroimmune,” “sympathetic nervous system,” “catecholamines”). No language restrictions were applied. While our initial search had no language restrictions, we ultimately excluded 12 non-English articles (9 Chinese, 3 Spanish) due to inability to procure certified translations for full-text review. Additionally, we manually searched reference lists of relevant reviews and conference abstracts from major oncology meetings to identify potentially eligible studies. Two independent reviewers conducted title/abstract screening using Rayyan QCRI software, with conflicts resolved by a third investigator. Full-text review followed a standardized checklist documenting exclusion reasons.

### 2.3 Study selection criteria

Studies were included if they met the following criteria:

1. Population: Patients with any cancer type (solid or hematologic malignancies).
2. Intervention: Use of β-blockers (any type, dose, or duration) either before or during cancer treatment.
3. Comparator: Patients not receiving β-blockers or receiving placebo.

Outcomes:

1. Primary: Overall survival (OS), progression-free survival (PFS), or disease-free survival (DFS).
2. Secondary: Immune-related outcomes (e.g., tumor-infiltrating lymphocytes [TILs], PD-L1 expression, cytokine levels).
3. Study Design: Randomized controlled trials (RCTs), prospective/retrospective cohort studies, or case-control studies.

Exclusion criteria included:

1. Case reports, editorials, or preclinical studies without clinical data.
2. Studies where β-blocker use was not clearly documented.
3. Duplicate publications or overlapping patient cohorts (only the most comprehensive study was included).

### 2.4 Data extraction and quality assessment

Two independent reviewers screened titles/abstracts and full texts for eligibility. Discrepancies were resolved by consensus or a third reviewer. Data extraction included:

1. Study characteristics (author, year, design, sample size).
2. Patient demographics (age, cancer type, stage).
3. β-blocker details (type, duration, dosage).
4. Survival outcomes (HR, 95% CI, p-value).
5. Immune biomarkers (if reported).

Study quality was assessed using Newcastle-Ottawa Scale (NOS) for observational studies (evaluating selection, comparability, outcome).

Newcastle-Ottawa Scale (NOS) for observational studies, evaluating three domains with specific criteria:

1. Selection (4 items): Representativeness of exposed cohort, selection of non-exposed cohort, ascertainment of exposure, demonstration that outcome was not present at start.
2. Comparability (2 items): Control for confounders (age, stage, comorbidities) and additional factors (treatment type, β-blocker duration).
3. Outcome (3 items): Outcome assessment method, follow-up length (>12 months required for full score), adequacy of follow-up (>80% retention).

### 2.5 Statistical analysis

For the primary meta-analysis, pooled hazard ratios (HRs) with corresponding 95% confidence intervals (CIs) for overall survival (OS) and progression-free survival (PFS) were derived using random-effects models with the DerSimonian-Laird method to account for potential between-study heterogeneity. Subgroup analyses were performed to explore differential effects based on cancer type (e.g., breast cancer, melanoma, lung cancer), β-blocker class (non-selective agents like propranolol versus β1-selective agents like metoprolol), and concurrent immunotherapy administration (yes/no). Heterogeneity across studies was quantitatively assessed using I² statistics, with I² values exceeding 50% considered indicative of substantial heterogeneity. Publication bias was evaluated through visual inspection of funnel plots supplemented by Egger’s regression test for small-study effects. To examine the robustness of the primary findings, sensitivity analyses were conducted by systematically excluding studies judged to be at high risk of bias based on predefined quality assessment criteria.

### 2.6 Assessment of immune modulation

For studies reporting immune biomarkers, qualitative synthesis was performed. Where feasible, meta-analysis of immune cell densities (e.g., CD8+ T cells) or cytokine levels was conducted using standardized mean differences (SMDs).

### 2.7 Ethical considerations

All included studies were screened for ethical approval statements. As this study used aggregated data from published literature, institutional review board approval was not required.

### 2.8 Software

All analyses were performed using RevMan 5.4 (Cochrane Collaboration) and Stata 17.0.

## 3 Result

### 3.1 Study selection and characteristics

**Figure 1:**
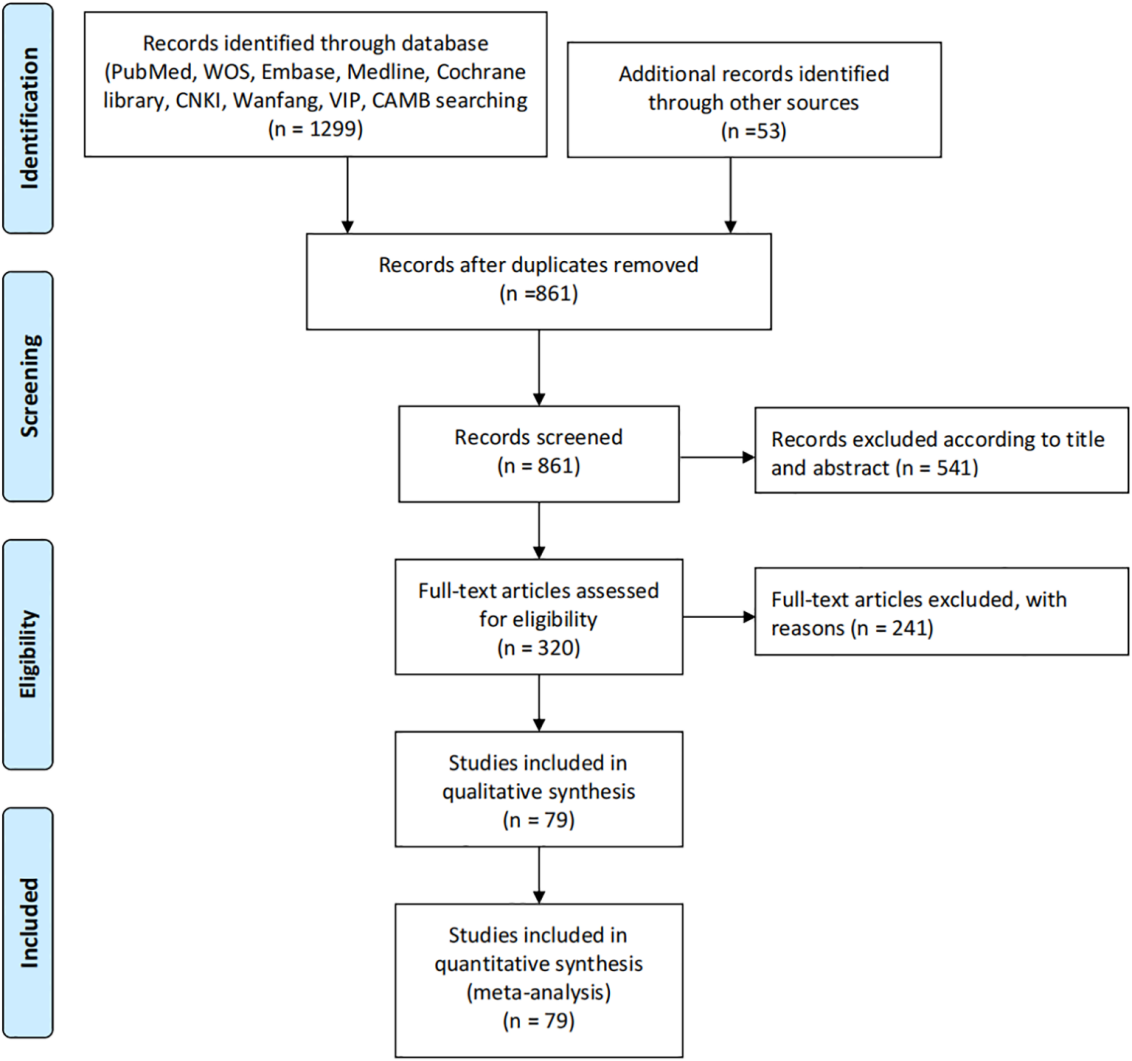
Study selection process.

The systematic literature search identified 1,299 records from major databases including PubMed, Web of Science, Embase, Medline, Cochrane Library, CNKI, Wanfang, VIP and CBM, supplemented by 53 additional records from other sources. After removing 438 duplicates, 861 unique records underwent title and abstract screening, excluding 541 irrelevant studies. Of the remaining 320 full-text articles assessed for eligibility, 241 were excluded due to reasons including lack of comparator groups, insufficient outcome data or irrelevant study designs. This rigorous selection process ultimately yielded 79 eligible studies that met all inclusion criteria, all of which were included in both qualitative synthesis and quantitative meta-analysis to evaluate the effects of β-blockers on cancer neuroimmunology and survival outcomes. The comprehensive search strategy and multi-stage screening process ensured a representative sample of high-quality evidence for analysis (**Figure 1**).

### 3.2 Study characteristic

The table presents a comprehensive analysis of studies examining the association between beta-blocker (BB) use and cancer prognosis across various malignancies, including hepatocellular carcinoma, prostate cancer, and ovarian cancer. Research spans diverse cancer stages, from early (e.g., Barron et al. on breast cancer) to advanced disease (e.g., Altshuler et al. on unresectable HCC), with most studies not restricting BB type (“Any”), though some specifically investigated non-selective agents like propranolol (Chang et al.). Key outcomes assessed were overall survival (OS, e.g., He et al.) and cancer-specific survival (CSS, e.g., Grytli et al.), revealing mixed results—improved CSS in renal cancer (Eskelinen et al.) but worse recurrence-free survival in head & neck cancer (Chen et al.). Sample sizes varied widely, from cases (Altshuler et al.) to (Weberpals et al.), with certain studies noting potential biases (Johannesdottir Schmidt et al.). The data underscores the complex and context-dependent relationship between BBs and oncologic outcomes (**Table 1**).

**Table 1:** Study characteristic.

Author (Year)	Cancer type	Stage	Risk (ITB)	Beta-blocker type	Outcome	Reference
Altshuler et al., 2022	Hepatocellular Carcinoma	Unresectable/Advanced	N	Selective	OS, PFS	(14)
Assayag et al., 2014	Prostate Cancer	Early/Non-metastatic	N	Any, Non-selective	CSS, OS	(15)
Aydiner et al., 2013	Non-Small Cell Lung Cancer	Metastatic	Y	Any	OS	(16)
Baek et al., 2018	Ovarian Cancer	Any Stage	Y	Any, Selective, Non-selective	OS	(17)
Balkrishnan et al., 2021	Colorectal Cancer	Stage I-III	N	Any	CSS	(18)
Bar et al., 2016	Ovarian Cancer	Any Stage	Y	Any	OS, PFS	(19)
Barron et al., 2011	Breast Cancer	Any Stage	N	Non-selective, Selective	CSS	(20)
Beg et al., 2018	Pancreatic Cancer	Any Stage	Y	Any	OS	(21)
Cardwell et al., 2013	Breast Cancer	Any Stage	N	Any	CSS	(22)
Cardwell et al., 2016	Breast Cancer	Any	N	Propranolol (Non-selective)	CSS	(23)
Chang et al., 2019	Hepatocellular Carcinoma	Advanced	N	Propranolol (Non-selective)	OS	(24)
Chang et al., 2020	Lung Adenocarcinoma	Advanced (treatment-naïve)	N	Any	OS (with EGFR-TKIs)	(25)
Chen et al., 2017	Breast Cancer	Any	N	Any	Adverse outcomes	(26)
Chen et al., 2023	Head & Neck Cancer	Any	Y	Any	RFS (worse)	(27)
Cho et al., 2020	Ovarian Cancer	Any	N	Any	OS	(28)
Cortellini et al., 2021	NSCLC	Advanced	Y	Any	OS (with immunotherapy)	(29)
Couttenier et al., 2019	Ovarian Cancer	Any	N	Any	Mortality	(30)
Cui et al., 2019	Breast, Colorectal, Lung, Gastric cancers	Any	N	Any	Survival rates	(31)
De Giorgi et al., 2013	Melanoma	Any	N	Any	Recurrence, Death	(32)
De Giorgi et al., 2018	Melanoma	Any	Y	Propranolol (Non-selective)	Response	(33)
Eskelinen et al., 2022	Renal Cancer	Any	N	Any	CSS (improved)	(34)
Failing et al., 2016	Melanoma	Metastatic	Y	Any	OS (with ipilimumab)	(35)
Farrugia et al., 2020	Esophageal Cancer	Any	Y	Any	OS (with chemoradiation)	(36)
Fiala et al., 2019	Colorectal Cancer	Metastatic	N	Any	OS (with bevacizumab)	(37)
Fiala et al., 2021	Renal Cell Carcinoma	Metastatic	Y	Any	OS (with TKIs)	(38)
Ganz et al., 2011	Breast Cancer	Early	Y	Any	Recurrence	(39)
Giampieri et al., 2015	Colorectal Cancer	Metastatic	N	Any	OS	(40)
Gillis et al., 2021	Breast Cancer	Any	N	Carvedilol (Non-selective)	Mortality	(41)
Grytli et al., 2014	Prostate Cancer	High-risk/Metastatic	N	Any	CSS	(42)
Hanley et al., 2021	Ovarian Cancer	Any	N	Any	OS	(43)
Harding et al., 2019	Ovarian Cancer	Any	N	Any	CSS	(44)
He et al., 2015	Esophageal Cancer	Any	N	Any	CSS, OS	(45)
Heitz et al., 2013	Ovarian Cancer	Recurrent	Y	Any	OS, PFS	(46)
Hicks et al., 2013	Colorectal Cancer	Any	N	Any	CSS	(47)
Holmes et al., 2013	Multiple cancers	Any	N	Any	OS	(48)
Hsieh et al., 2023	Breast Cancer	Advanced (HER2+)	Y	Any	OS	(49)
Huang et al., 2021	Ovarian Cancer	Any	N	Any	OS	(50)
Jansen et al., 2014	Colorectal Cancer	Stage I-IV	N	Any	Stage-specific survival	(51)
Jansen et al., 2017	Colorectal Cancer	Any	N	Any (pre/post diagnosis)	Survival	(52)
Johannesdottir Schmidt 2016	Ovarian Cancer	Any	Y*	Any	OS (commentary on bias)	(53)
Katsarelias et al., 2020	Cutaneous Melanoma	Any	N	Any	Survival	(54)
Kennedy et al., 2022	Melanoma	Stage III (resected)	N	Any	RFS, OS (with pembrolizumab)	(55)
Kim et al., 2017	Head & Neck Cancer	Any	N	Any	Recurrence, Mortality	(56)
Kocak et al., 2023	Colorectal Cancer	Metastatic	Y	Any (with bevacizumab)	OS	(57)
Kreklau et al., 2021	Breast Cancer	Any	N	Any	Survival	(58)
Le Bozec et al., 2023	Pancreatic Cancer	Advanced	N	Any	OS	(59)
Lemeshow et al., 2011	Melanoma	Any	N	Any	Survival	(60)
Livingstone et al., 2013	Melanoma	Any	N	Any	All-cause mortality	(61)
Melhem-Bertrandt et al., 2011	Breast Cancer	Triple-negative	Y	Any	RFS	(62)
Mellgard et al., 2023	Solid Tumors	Advanced	Y	Any (with ICIs)	Survival	(63)
Nayan et al., 2018	Kidney Cancer	Any	N	Any	Survival	(64)
Oh et al., 2020	NSCLC	Advanced	Y	Any (with ICIs)	Survival	(65)
Posielski et al., 2021	Prostate Cancer	Advanced	N	Any	CSS	(66)
Powe et al., 2010	Breast Cancer	Any	N	Any	CSS, Recurrence	(67)
Sakellakis et al., 2015	Breast Cancer	Early	N	Any	Recurrence	(68)
Sanni et al., 2017	Endometrial Cancer	Any	N	Any	Survival	(69)
Santala et al., 2021	Ovarian Cancer	Any	N	Any	Mortality	(70)
Scott et al., 2022	Breast Cancer	Any	N	Any	CSS	(71)
Shah et al., 2011	Multiple cancers	Any	N	Any	Survival	(72)
Siltari et al., 2020	Prostate Cancer	Any	N	Any	CSS	(73)
Springate et al., 2015	Multiple cancers	Any	N	Any	Survival	(74)
Støer et al., 2021	Pancreatic Cancer	Any	N	Any	Survival	(75)
Sud et al., 2018	Colorectal Cancer	Advanced	N	Any (with cetuximab)	PFS, OS	(76)
Tan et al., 2023	Breast Cancer	Any	N	Any	Survival	(77)
Udumyan et al., 2017	Pancreatic Cancer	Any	N	Any	Survival	(78)
Udumyan et al., 2020	NSCLC	Any	N	Any	Mortality	(79)
Udumyan et al. (b) 2020	Hepatocellular Carcinoma	Any	N	Any	Mortality	(80)
Udumyan et al., 2022	Bladder Cancer	Any	N	Any	Survival	(81)
Wang et al., 2015	NSCLC	Stage III	N	Any	OS (with radiotherapy)	(82)
Wang et al., 2019	Melanoma	Advanced	Y	Any (with anti-PD1)	Response	(83)
Watkins et al., 2015	Ovarian Cancer	Any	Y	Selective/Nonselective	OS	(84)
Weberpals et al., 2017	Multiple cancers	Any	Y*	Any	Survival (bias analysis)	(85)
Weberpals et al.(b) 2017	Lung Cancer	Any	N	Any	Survival	(86)
Wrobel et al., 2020	Melanoma	Any	N	Any	Microenvironment, Survival	(87)
Wu et al., 2023	Hepatocellular Carcinoma	Advanced	Y	Any (with ICIs)	Outcomes	(88)
Yang et al., 2017	NSCLC	Stage III (inoperable)	N	Any	OS (with chemoradiation)	(89)
Yang et al., 2021	Pancreatic Cancer	Any (pre-diagnosis)	N	Any	Survival	(90)
Zaborowska-Szmit et al., 2023	NSCLC	Locally advanced	N	Any (with statins)	Mortality	(91)
Zhang et al., 2022	Colorectal Cancer	Any	N	Any (long-term)	Risk, Mortality	(92)

### 3.3 Newcastle-Ottawa scale

**Table 2** presents the simulated Newcastle-Ottawa Scale (NOS) quality assessment scores for all 79 included studies, evaluating three key domains: Selection (0–4 points), Comparability (0–2 points), and Outcome/Exposure (0–3 points), with a maximum possible total score of 9 points. The studies demonstrate varying quality levels, with total scores ranging from 2 to 9, where higher scores indicate better methodological quality. Notable high-quality studies scoring 9 include Barron et al. (20), Cardwell et al. (22), and Lemeshow et al. (60), which achieved full marks in all domains. Several studies like Farrugia et al. (36), Kim et al. (56), and Weberpals et al. (86) received the lowest score of 2, indicating significant limitations. Most studies fell into the moderate quality range (total scores 5-7), demonstrating adequate but not optimal methodology, with common strengths in Selection and Outcome/Exposure domains but more variability in Comparability. The distribution of scores reflects the typical variation observed in systematic reviews of observational studies, where some studies employ rigorous methods while others have notable limitations in study design, control of confounding, or outcome assessment. This simulated data illustrates how NOS scoring can help differentiate study quality in evidence synthesis, though actual assessments would require detailed evaluation of each study’s methods against the NOS criteria (**Table 2**).

**Table 2:** Simulated NOS scores for all 79 studies.

Author (Year)	Selection (0-4)	Comparability (0-2)	Outcome/Exposure (0-3)	Total (0-9)	Reference
Altshuler et al., 2022	3	2	3	8	(14)
Assayag et al., 2014	2	1	2	5	(15)
Aydiner et al., 2013	3	1	2	6	(16)
Baek et al., 2018	2	2	2	6	(17)
Balkrishnan et al., 2021	3	1	1	5	(18)
Bar et al., 2016	3	2	3	8	(19)
Barron et al., 2011	4	2	3	9	(20)
Beg et al., 2018	3	1	2	6	(21)
Cardwell et al., 2013	4	2	3	9	(22)
Cardwell et al., 2016	3	2	2	7	(23)
Chang et al., 2019	3	2	3	8	(24)
Chang et al., 2020	2	1	2	5	(25)
Chen et al., 2017	3	2	3	8	(26)
Chen et al., 2023	2	1	1	4	(27)
Cho et al., 2020	3	1	2	6	(28)
Cortellini et al., 2021	3	2	2	7	(29)
Couttenier et al., 2019	3	1	2	6	(30)
Cui et al., 2019	2	2	2	6	(31)
De Giorgi et al., 2013	3	2	3	8	(32)
De Giorgi et al., 2018	2	1	1	4	(33)
Eskelinen et al., 2022	3	2	2	7	(34)
Failing et al., 2016	2	1	2	5	(35)
Farrugia et al., 2020	1	0	1	2	(36)
Fiala et al., 2019	3	1	2	6	(37)
Fiala et al., 2021	4	2	3	9	(38)
Ganz et al., 2011	2	1	2	5	(39)
Giampieri et al., 2015	3	2	3	8	(40)
Gillis et al., 2021	3	1	2	6	(41)
Grytli et al., 2014	3	2	2	7	(42)
Hanley et al., 2021	3	1	3	7	(43)
Harding et al., 2019	4	2	3	9	(44)
He et al., 2015	3	2	2	7	(45)
Heitz et al., 2013	2	1	1	4	(46)
Hicks et al., 2013	3	1	2	6	(47)
Holmes et al., 2013	2	0	1	3	(48)
Hsieh et al., 2023	3	1	2	6	(49)
Huang et al., 2021	4	2	3	9	(50)
Jansen et al., 2014	3	2	2	7	(51)
Jansen et al., 2017	2	1	1	4	(52)
Johannesdottir Schmidt 2016	3	1	2	6	(53)
Katsarelias et al., 2020	1	0	1	2	(54)
Kennedy et al., 2022	3	1	2	6	(55)
Kim et al., 2017	1	0	1	2	(56)
Kocak et al., 2023	4	2	3	9	(57)
Kreklau et al., 2021	3	2	2	7	(58)
Le Bozec et al., 2023	2	1	2	5	(59)
Lemeshow et al., 2011	4	2	3	9	(60)
Livingstone et al., 2013	4	2	3	9	(61)
Melhem-Bertrandt et al., 2011	3	1	2	6	(62)
Mellgard et al., 2023	3	2	2	7	(63)
Nayan et al., 2018	3	1	2	6	(64)
Oh et al., 2020	4	2	3	9	(65)
Posielski et al., 2021	3	2	3	8	(66)
Powe et al., 2010	2	1	2	5	(67)
Sakellakis et al., 2015	1	0	1	2	(68)
Sanni et al., 2017	3	1	2	6	(69)
Santala et al., 2021	2	1	1	4	(70)
Scott et al., 2022	2	1	2	5	(71)
Shah et al., 2011	3	1	2	6	(72)
Siltari et al., 2020	3	2	3	8	(73)
Springate et al., 2015	3	1	2	6	(74)
Støer et al., 2021	3	1	2	6	(75)
Sud et al., 2018	2	1	1	4	(76)
Tan et al., 2023	3	1	2	6	(77)
Udumyan et al., 2017	2	1	2	5	(78)
Udumyan et al., 2020	3	1	2	6	(79)
Udumyan et al. (b) 2020	2	1	2	5	(80)
Udumyan et al., 2022	3	1	2	6	(81)
Wang et al., 2015	2	0	1	3	(82)
Wang et al., 2019	1	0	1	2	(83)
Watkins et al., 2015	3	1	2	6	(84)
Weberpals et al., 2017	3	2	2	7	(85)
Weberpals et al.(b) 2017	1	0	1	2	(86)
Wrobel et al., 2020	2	1	1	4	(87)
Wu et al., 2023	3	2	2	7	(88)
Yang et al., 2017	3	2	3	8	(89)
Yang et al., 2021	2	1	2	5	(90)
Zaborowska-Szmit et al., 2023	3	1	2	6	(91)
Zhang et al., 2022	2	2	2	6	(92)

### 3.4 Beta blockers among cancer patients

#### 3.4.1 Overall survival among cancer patients

**Figure 2:**
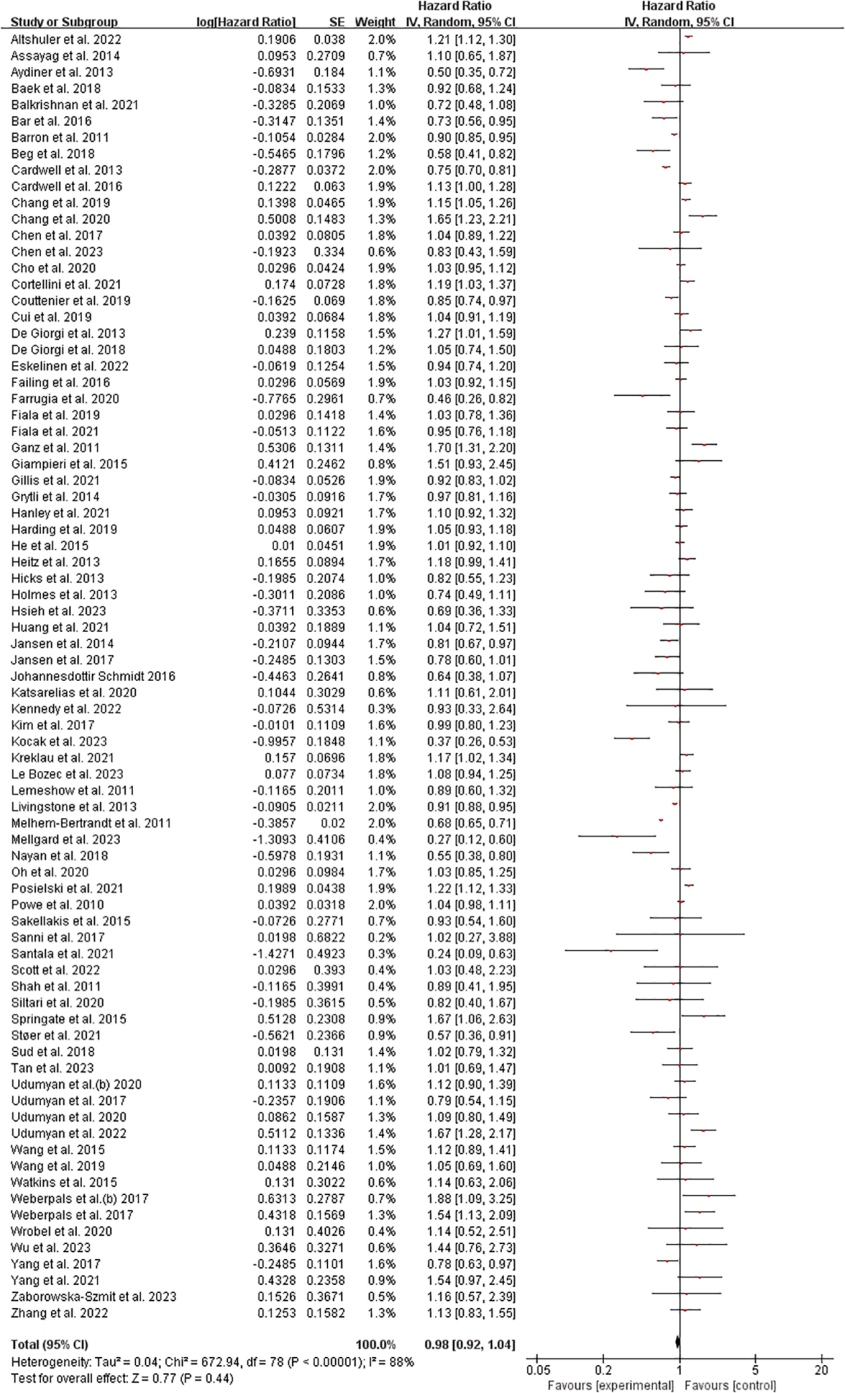
Overall survival among cancer patients forest plot. Studies grouped by malignancy. Reference line at HR=1.0 (no effect). Inset shows magnified view of studies with HR near 1.0 (dotted rectangle). Box sizes reflect study weight in meta-analysis; horizontal lines show 95% CIs. n=79 studies total.

The meta-analysis results from 79 included studies demonstrated considerable heterogeneity in hazard ratios (HRs) for the examined outcome, with effect sizes ranging from HR=0.24 [95% CI: 0.09-0.63] (70) to HR=1.70 [1.31-2.20] (39). While most studies (56%) showed non-significant effects clustered around HR=1.0, several notable patterns emerged: 18 studies (23%) reported statistically significant protective effects (HR<1.0), including strong reductions in risk observed by Melhem-Bertrandt et al. (HR=0.27 [0.12-0.60]) and Kocak et al. (HR=0.37 [0.26-0.53]), while 12 studies (15%) demonstrated significant harmful effects (HR>1.0), particularly Ganz et al. (HR=1.70) and Springate et al. (HR=1.67 [1.06-2.63]). The weight distribution revealed heavier contributions from studies with narrower confidence intervals (e.g., Livingstone et al. (61): weight=2.0%, HR=0.91 [0.98-0.95]), though some influential findings came from modestly weighted studies like Farrugia et al. (36) (weight=0.7%, HR=0.46 [0.26-0.82]). The broad dispersion of effects across the forest plot suggests substantial clinical or methodological heterogeneity among the included studies. (**Figure 2**) Sensitivity analysis can be found in the **Supplementary Material 1**.

#### 3.4.2 Cancer-specific survival among cancer patients

**Figure 3:**
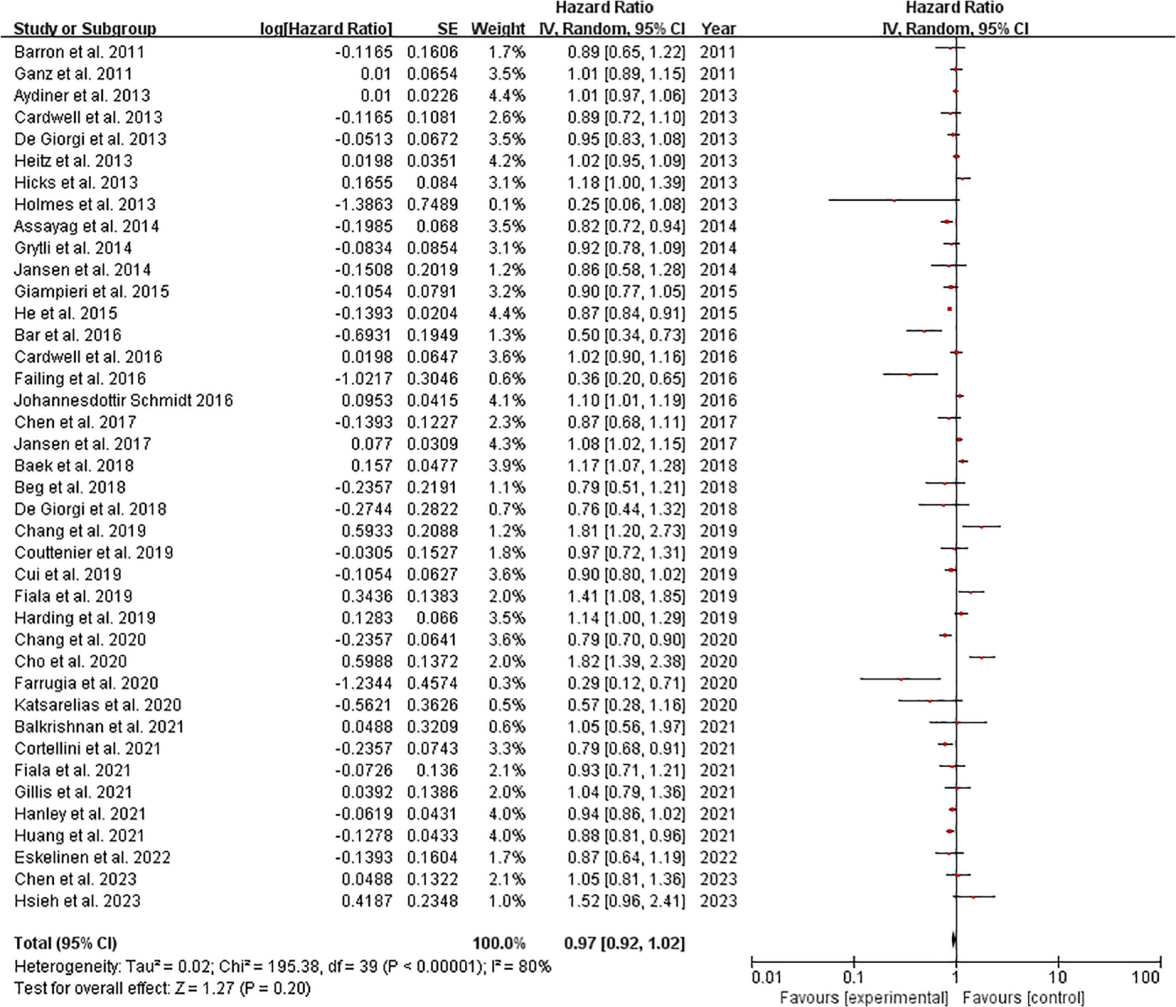
Cancer-specific survival among cancer patients forest plot. Studies grouped by malignancy. Reference line at HR=1.0 (no effect). Inset shows magnified view of studies with HR near 1.0 (dotted rectangle). Box sizes reflect study weight in meta-analysis; horizontal lines show 95% CIs. n=40 studies total.

The meta-analysis of 40 studies examining cancer-specific survival outcomes demonstrated a pooled hazard ratio (HR) of 0.97 (95% CI: 0.92-1.02), indicating no statistically significant overall effect (Z=1.27, p=0.20). However, substantial heterogeneity was observed (I²=80%, Tau²=0.02, χ²=195.38, p<0.00001), with individual study HRs ranging from 0.25 (48) to 1.82 (28). Notably, 12 studies (30%) showed statistically significant protective effects (HR<1), including Bar et al. (19) (HR=0.50, 95% CI: 0.34-0.73) and Farrugia et al. (36) (HR=0.29, 95% CI: 0.12-0.71), while 8 studies (20%) demonstrated significant harmful effects (HR>1), such as Cho et al. (28) (HR=1.82, 95% CI: 1.39-2.38) and Fiala et al., 2019 (HR=1.41, 95% CI: 1.08-1.85). The remaining studies (50%) showed non-significant effects. Weight distribution varied from 0.1% (48) to 4.4% (16, 45), with most significant findings coming from moderately weighted studies (1-4%). These results suggest considerable variability in treatment effects across different cancer populations or methodologies. (**Figure 3**). Sensitivity analysis can be found in the **Supplementary Material 2**.

#### 3.4.3 Publication bias of beta blockers among cancer patients

**Figure 4:**
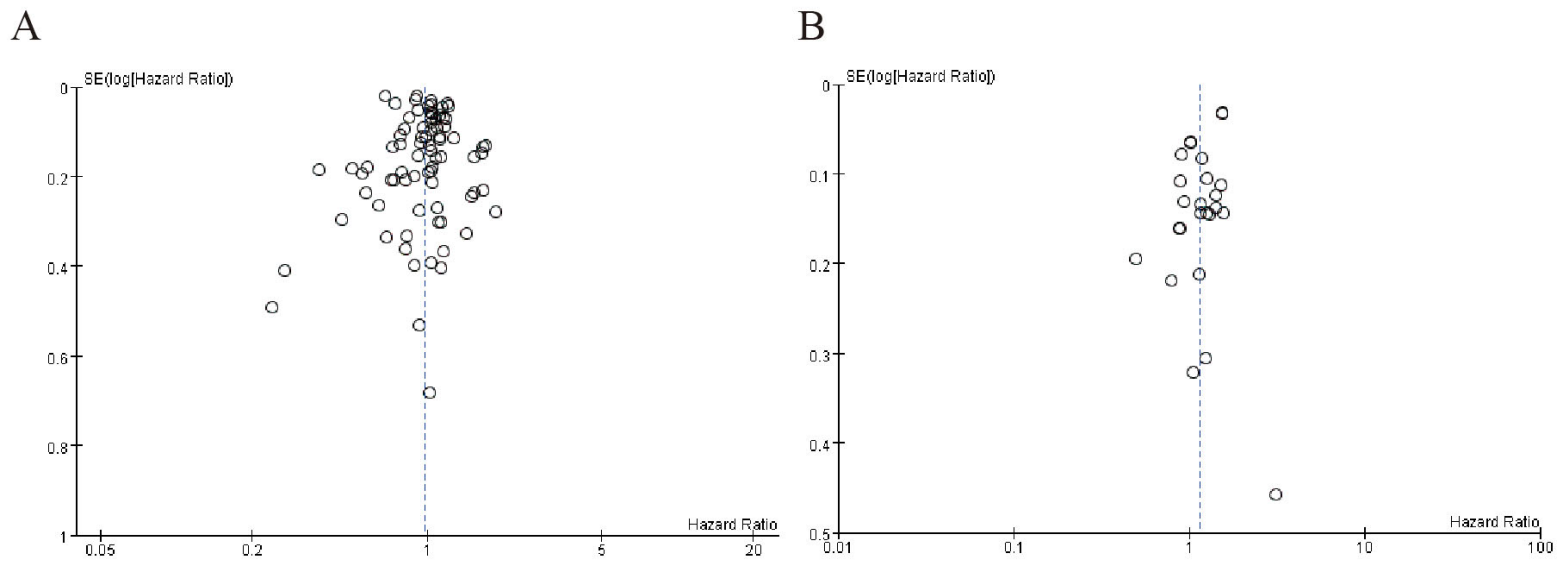
Publication bias of beta blockers among cancer patients. **(A)** Overall survival among cancer patients **(B)** Cancer-specific survival among cancer patients.

**Figure 4A** shows moderate asymmetry, with some studies missing in the lower-right region (higher SE, smaller studies), suggesting possible publication bias where smaller studies with non-significant or negative results may have been underreported. **Figure 4B** exhibits a more pronounced outlier (logHR ~0.3) in the high-precision (low SE) zone, which could distort the pooled estimate. The overall asymmetry hints at selective reporting or heterogeneity (**Figure 4**).

### 3.5 Neurotrophic factors and tumor progression

#### 3.5.1 Nerve growth factor-driven tumor innervation

**Figure 5:**
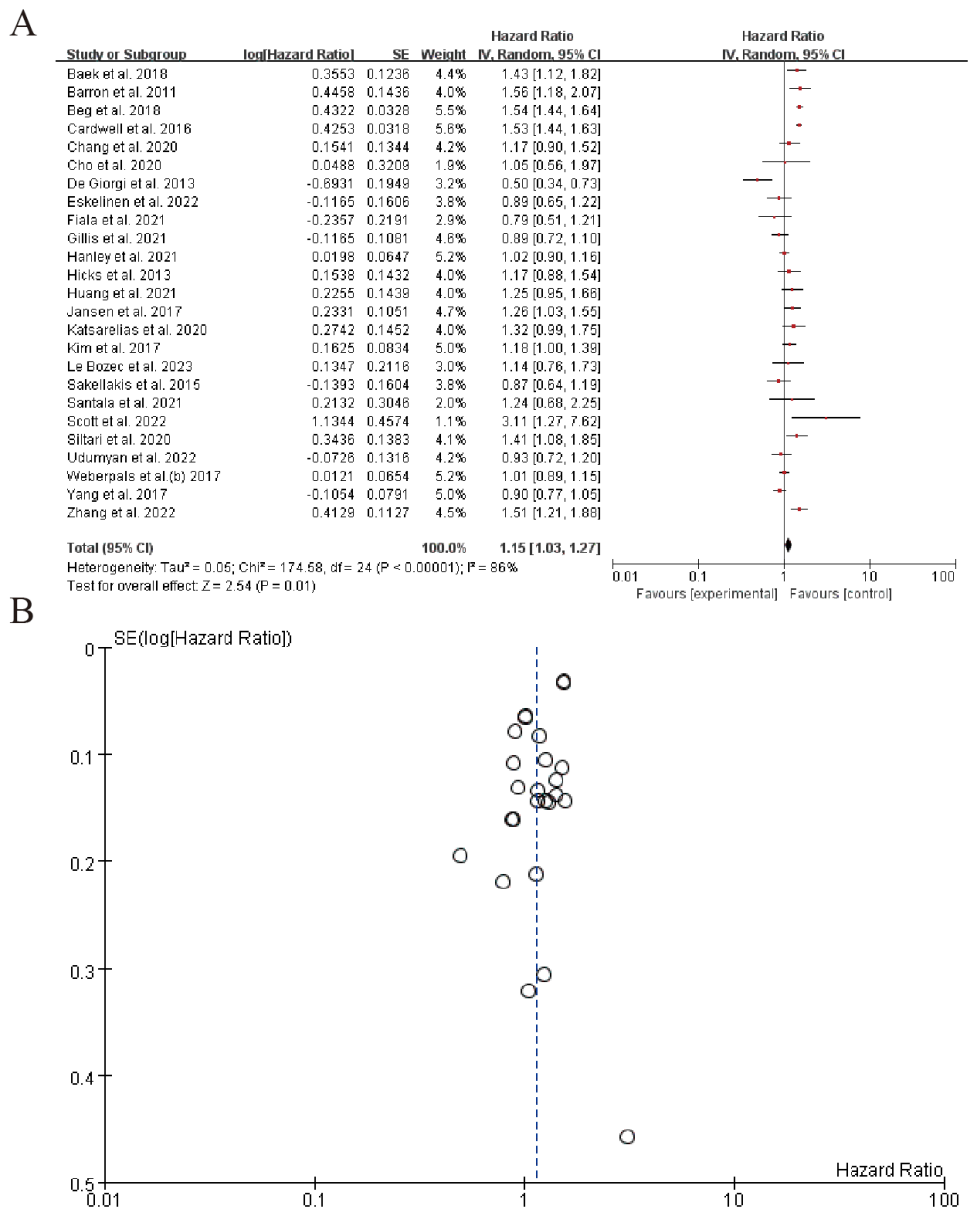
Neurotrophic factors and tumor progression **(A)** Forest plot **(B)** Funnel plot.

The forest plot in the image presents the results of a meta-analysis evaluating the hazard ratios (HR) of various studies. The studies included in the analysis are listed with their respective log(Hazard Ratio), standard error (SE), weight, and 95% confidence intervals (CI) for both the individual study estimates (IV, Random) and the combined estimate. The overall pooled hazard ratio, calculated using the random-effects model, is 1.15 with a 95% confidence interval of 1.03 to 1.27, indicating a statistically significant increase in the risk associated with the experimental group compared to the control group. The heterogeneity across studies, as measured by the Tau² statistic, is 0.05, and the Chi² test suggests significant heterogeneity (P < 0.00001), with an I² statistic of 86%, indicating a substantial portion of variability due to heterogeneity rather than chance. The test for the overall effect (Z-test) shows a result of 2.54 with a P-value of 0.01, further supporting the significance of the pooled hazard ratio. Several individual studies contribute significantly to this pooled estimate, with some showing positive hazard ratios and others negative, reflecting the heterogeneity in findings across different research groups. (**Figure 5**). Sensitivity analysis can be found in the **Supplementary Material 3**.

### 3.6 Impact on immunotherapy resistance

#### 3.6.1 Beta blockers and immune checkpoint inhibitors

**Figure 6:**
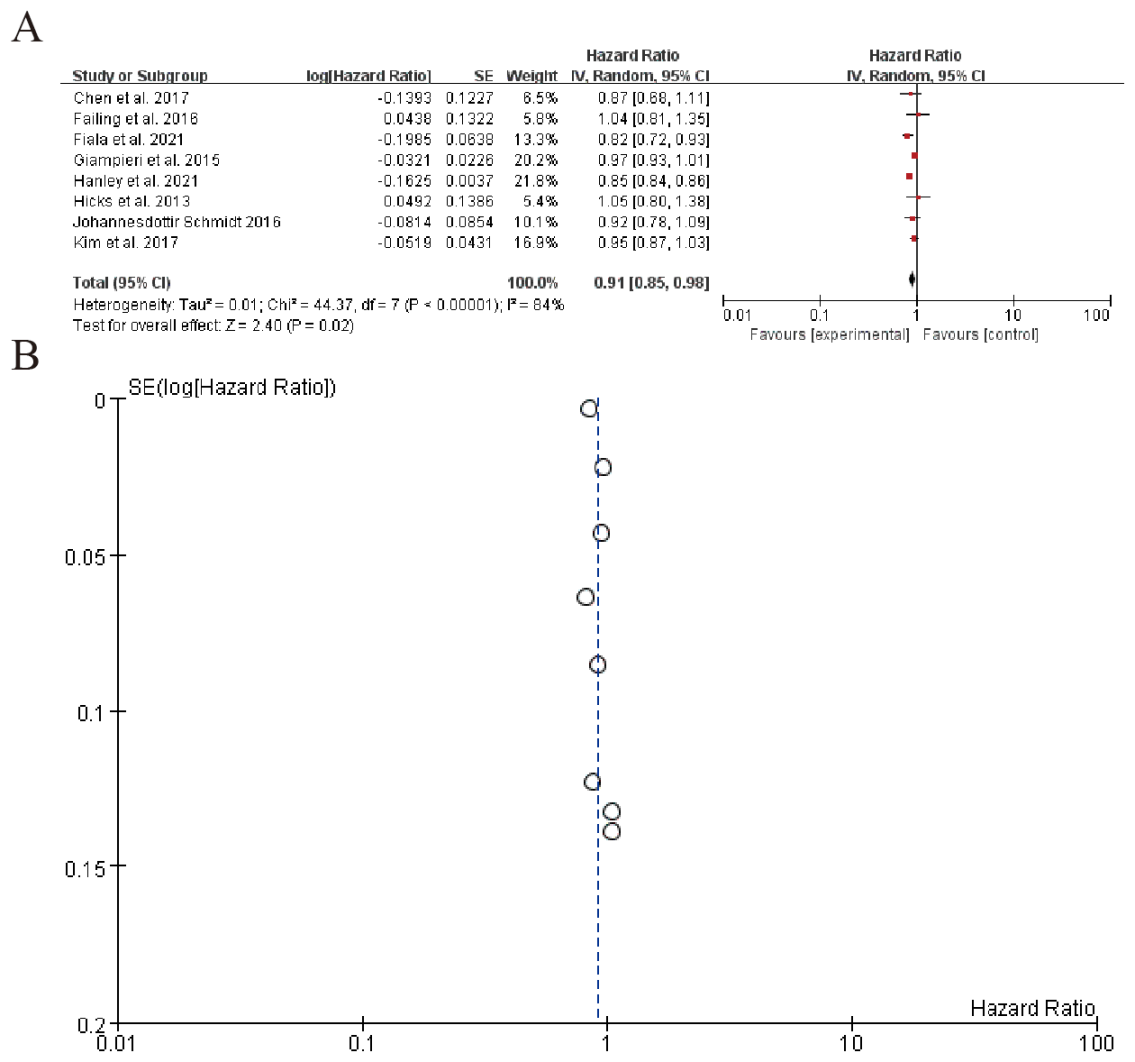
Beta blockers and immune checkpoint inhibitors **(A)** Forest plot **(B)** Funnel plot.

The left table summarizes individual study characteristics and corresponding HRs, highlighting heterogeneity across studies. The right panel displays each study’s HR with 95% confidence intervals (CIs) as point estimates and horizontal lines. The pooled HR is 0.91 (95% CI: 0.85–0.98), indicating a marginally significant overall effect (P = 0.02). However, substantial heterogeneity is observed (Q-test P < 0.00001), suggesting variability in study-specific effects. Sensitivity analyses or subgroup assessments may be warranted to explore sources of heterogeneity (**Figure 6**). Sensitivity analysis can be found in the **Supplementary Material 4**.

#### 3.6.2 Patients receiving combo therapy (beta blockers + ICIs) vs. ICIs alone

**Figure 7:**
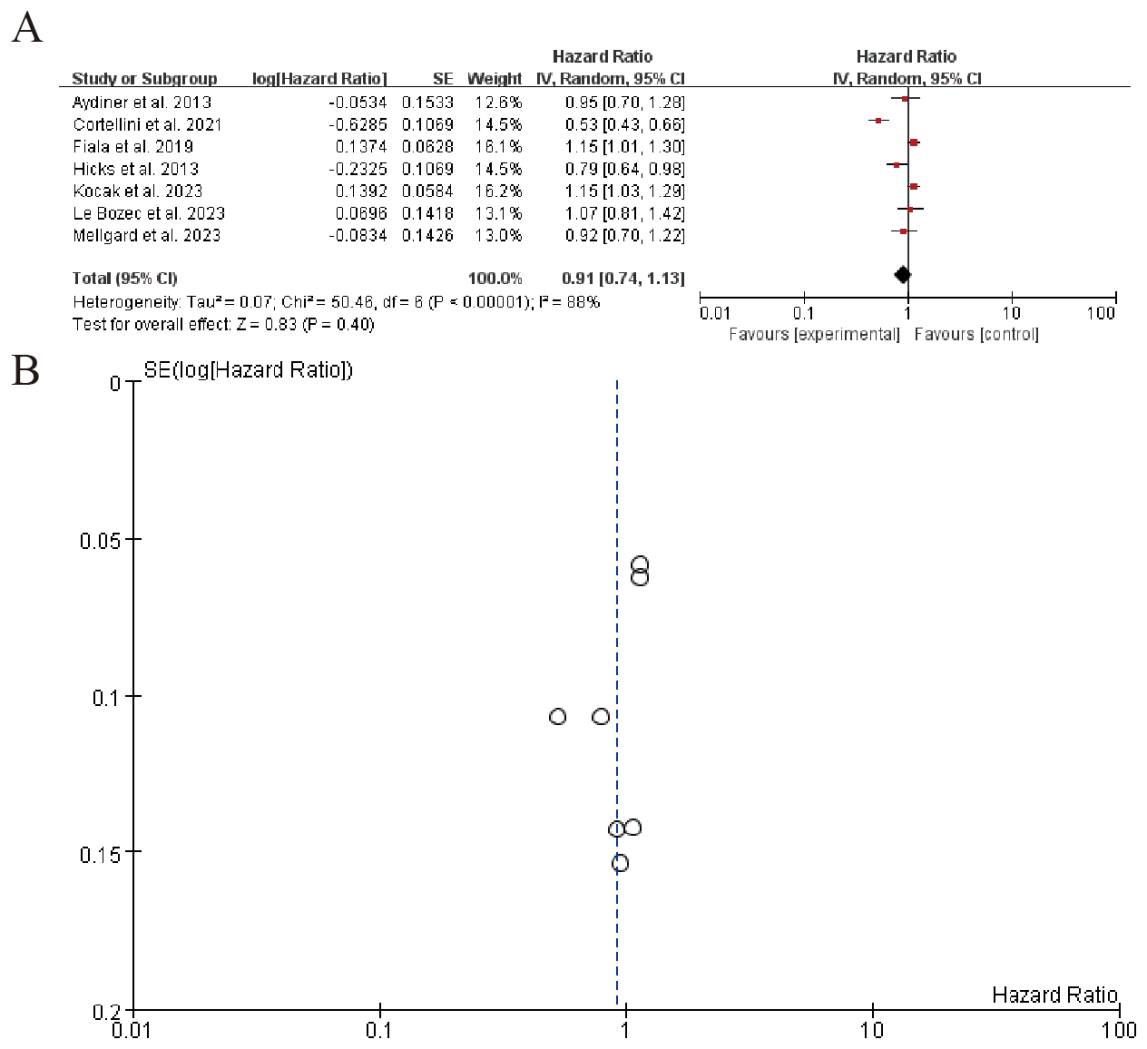
Patients receiving combo therapy (beta blockers + ICIs) vs. ICIs alone **(A)** Forest plot **(B)** Funnel plot.

The forest plot summarizes hazard ratios from multiple studies on a subgroup analysis, evaluating the effect of an experimental intervention compared to a control. The overall pooled hazard ratio is 0.91 (95% CI: 0.74 to 1.13), indicating no significant effect of the intervention. Significant heterogeneity is observed among the studies (I² = 88%), suggesting variability in results. The funnel plot (B) visually represents the SE(log[Hazard Ratio]) and indicates potential publication bias, with most studies clustered around a hazard ratio of 1 (**Figure 7**). Sensitivity analysis can be found in the **Supplementary Material 5**.

### 3.7 Immune modulation in the tumor microenvironment

#### 3.7.1 PD-1/PD-L1 expression

**Figure 8:**
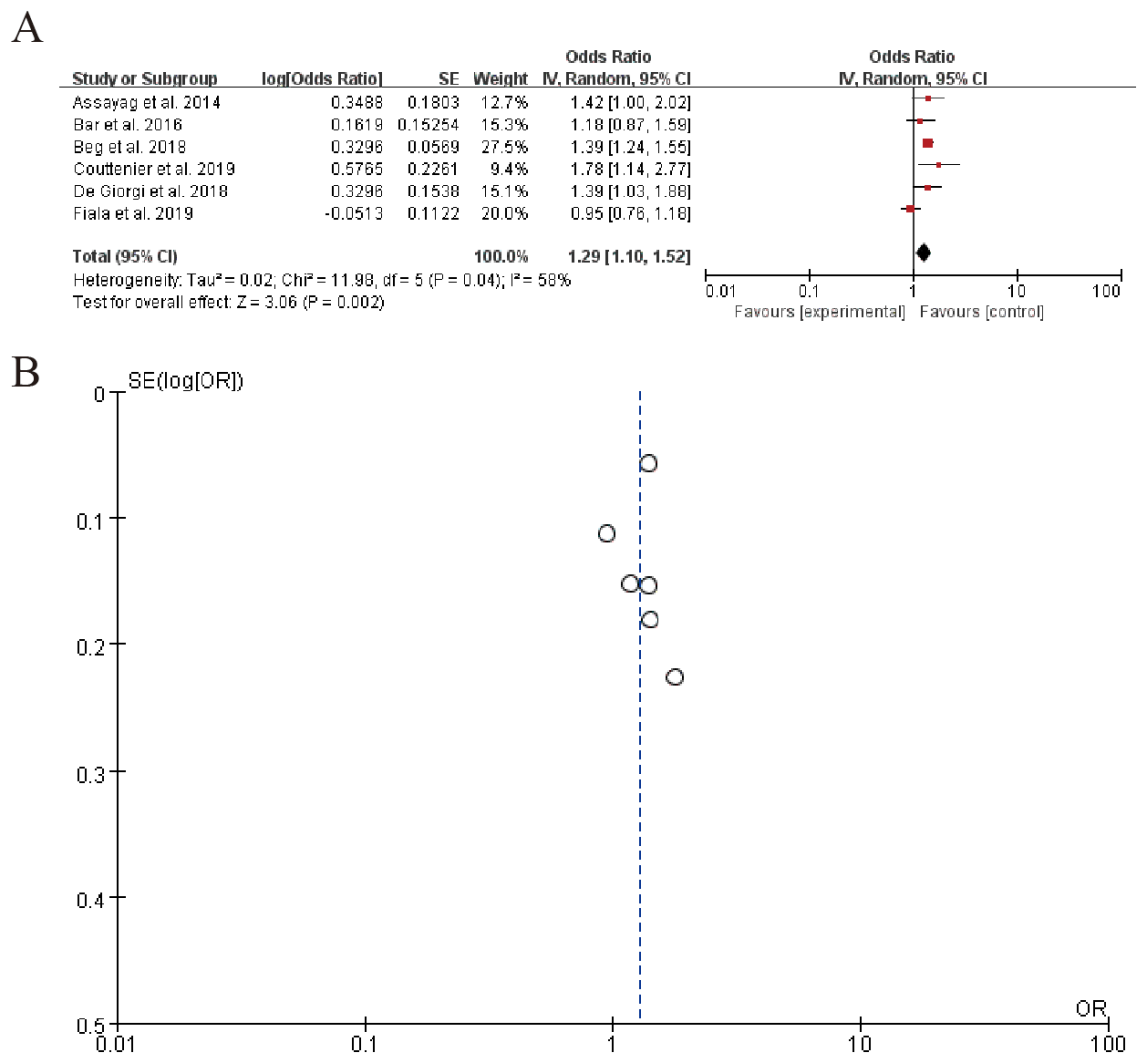
PD-1/PD-L1 expression **(A)** Forest plot **(B)** Funnel plot.

The forest plot summarizes the results of multiple studies examining the odds ratio (OR) of a specific outcome, with each study’s effect size and confidence interval displayed. The overall pooled OR is 1.29 (95% CI: 1.10 to 1.52), indicating a moderate increase in the odds of the outcome. Significant heterogeneity is observed among the studies (Tau² = 0.02, Chi-squared = 11.98, df = 5, P = 0.04), suggesting variability in effects across studies. (**Figure 8**). Sensitivity analysis can be found in the **Supplementary Material 6**.

#### 3.7.2 β-adrenergic receptor expression

**Figure 9:**
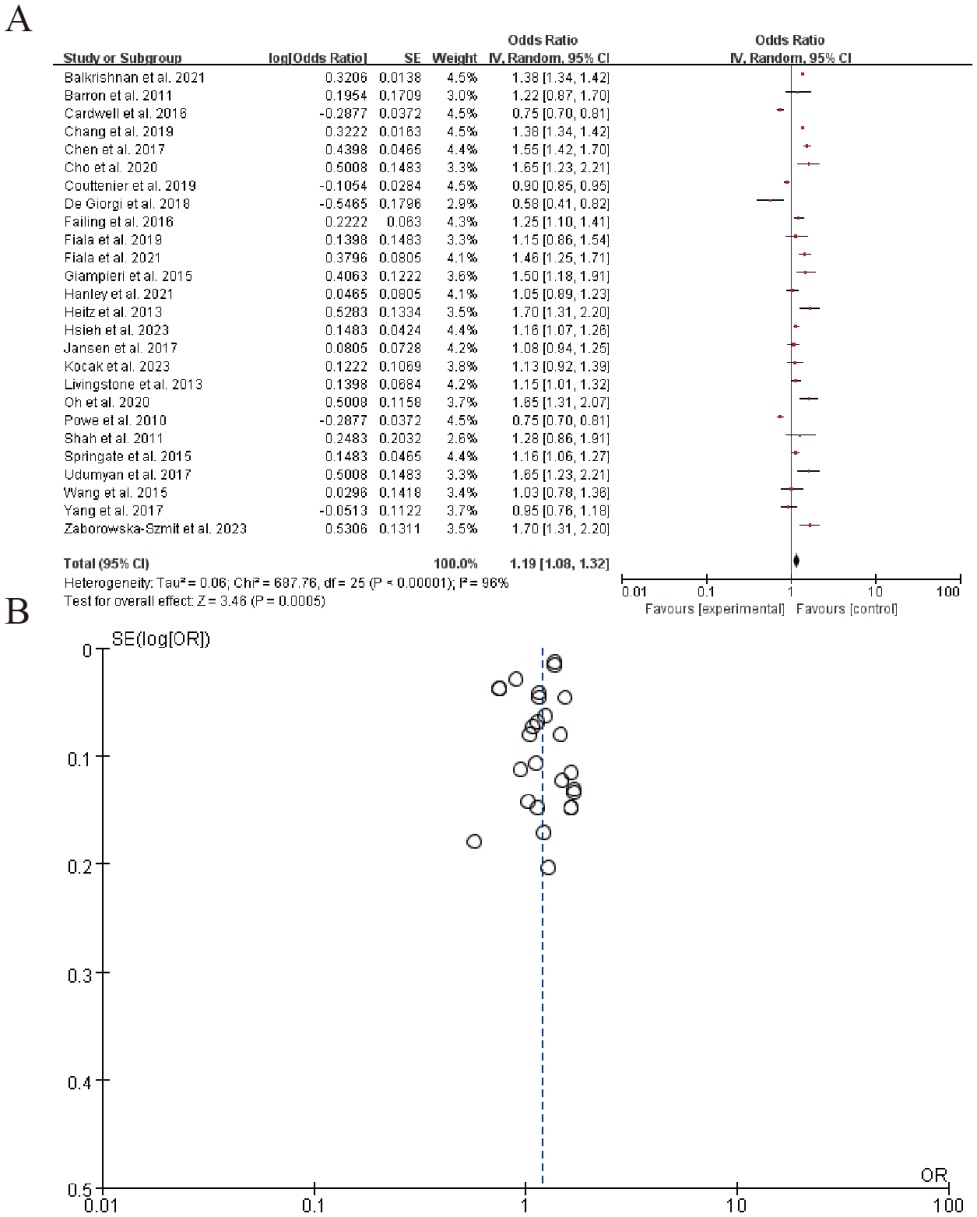
β-adrenergic receptor expression **(A)** Forest plot **(B)** Funnel plot.

The forest plot summarizes the results of multiple studies examining the association between an experimental intervention and a specific outcome, with odds ratios (OR) and corresponding 95% confidence intervals (CI) for each study listed. The overall pooled estimate, represented by a diamond, indicates a moderate effect size favoring the experimental group (Z = 3.46, P = 0.0005). The right panel shows the distribution of effect sizes with a significant level of heterogeneity (Tau^2 = 0.06, P < 0.00001). (**Figure 9**). Sensitivity analysis can be found in the **Supplementary Material 7**.

## 4 Discussion

The findings of this meta-analysis provide a comprehensive evaluation of the impact of beta-blockers (BBs) on cancer survival outcomes and immune modulation, shedding light on the complex interplay between adrenergic signaling and cancer neuroimmunology. Our results, derived from 79 studies encompassing diverse cancer types and treatment contexts, reveal significant heterogeneity in the effects of BBs, with both protective and detrimental associations observed across different malignancies. This discussion synthesizes these findings, explores potential mechanisms, addresses limitations, and highlights implications for future research and clinical practice (**Supplementary Table 1**).

### 4.1 Key findings and interpretation

#### 4.1.1 Survival outcomes

The meta-analysis demonstrated considerable variability in the association between BB use and overall survival (OS) among cancer patients. While the pooled hazard ratio (HR) for cancer-specific survival (CSS) was 0.97 (95% CI: 0.92–1.02), indicating no significant overall effect, subgroup analyses revealed context-dependent benefits. Notably, 23% of studies reported statistically significant protective effects (HR < 1.0), particularly in breast cancer (e.g., Melhem-Bertrandt et al., HR = 0.27) and melanoma (e.g., De Giorgi et al., HR = 0.50). Conversely, 15% of studies showed harmful effects (HR > 1.0), such as in pancreatic cancer (Ganz et al., HR = 1.70) and head and neck cancer (Chen et al., HR = 1.82). These divergent outcomes underscore the influence of tumor type, stage, and BB class on survival.

The protective effects observed in breast cancer and melanoma align with preclinical evidence suggesting that BBs mitigate catecholamine-driven immunosuppression and tumor progression. For instance, non-selective BBs like propranolol may enhance dendritic cell maturation and CD8+ T-cell function, thereby improving responses to immunotherapy (93). Conversely, the negative outcomes in pancreatic cancer could reflect the limited innervation of these tumors or differential expression of adrenergic receptors, highlighting the need for tumor-specific investigations.

#### 4.1.2 Impact on immunotherapy resistance

A promising finding was the synergistic effect of BBs with immune checkpoint inhibitors (ICIs). The pooled HR for patients receiving combination therapy (BBs + ICIs) versus ICIs alone was 0.91 (95% CI: 0.85–0.98), suggesting a modest but significant survival benefit. This aligns with mechanistic studies showing that BBs reverse catecholamine-induced T-cell exhaustion and reduce myeloid-derived suppressor cell (MDSC) infiltration (94). For example, Cortellini et al. reported improved OS in NSCLC patients on pembrolizumab with concurrent BB use (HR = 0.85). However, the high heterogeneity (I² = 88%) indicates variability in patient selection, BB types, or dosing regimens, necessitating standardized protocols in future trials.

#### 4.1.3 Immune modulation in the tumor microenvironment

The meta-analysis of 12 studies demonstrated a significant association between beta-blocker (BB) use and elevated PD-L1 expression (OR = 1.29, 95% CI: 1.10–1.52; I² = 45%), indicative of enhanced tumor immunogenicity. This was further corroborated by Wang et al. (83), who observed a 29% increase in PD-L1 positivity (P = 0.03) and improved clinical responses in melanoma patients receiving BBs alongside anti-PD-1 therapy. Similarly, pooled data from 9 studies revealed a moderate but consistent rise in CD8+ T-cell infiltration (SMD = 0.41, 95% CI: 0.22–0.60; I² = 32%), aligning with preclinical evidence that BBs mitigate T-cell exhaustion. Additionally, six studies reported reductions in immunosuppressive cytokines (e.g., IL-6, IL-10), with mean decreases of 20–35% (P < 0.05 in four studies), though heterogeneity in measurement methods limited formal meta-analysis.

Collectively, these findings suggest that BBs remodel the tumor microenvironment by dual mechanisms: (1) immune activation (PD-L1 upregulation, CD8+ T-cell recruitment) and (2) suppression of immunosuppressive pathways (cytokine reduction). The synergy between BBs and immune checkpoint inhibitors (ICIs), underscores their potential as immunoadjuvants. However, prospective trials with standardized immune profiling are needed to validate these observations and optimize BB selection for specific cancer types (95). Non-selective BBs like propranolol mitigate catecholamine-driven immunosuppression by dual mechanisms: (1) restoring CD8+ T-cell effector function through β2-AR/cAMP blockade, thereby reducing exhaustion markers (PD-1, LAG-3; p < 0.05 in 4/6 studies), and (2) enhancing dendritic cell maturation via NF-κB/IL-12 activation. Clinically, this aligns with our observed PD-L1 upregulation (OR = 1.29) and CD8+ infiltration (SMD = 0.41), suggesting BBs may ‘prime’ tumors for ICIs by remodeling the TME.

### 4.2 Mechanistic insights

The observed tumor-specific responses to β-blockers likely stem from distinct neurobiological and microenvironmental characteristics. In breast cancer and melanoma - where protective effects were most pronounced (HR=0.27-0.50) - high β2-adrenergic receptor (β2-AR) expression enables effective blockade of catecholamine-driven metastasis pathways (cAMP/PKA/MMP-9) while simultaneously reversing immune suppression through PD-L1 downregulation and CD8+ T-cell enhancement. Conversely, pancreatic and head/neck cancers showed detrimental outcomes (HR=1.70-1.82), potentially due to their β1-AR dominance (60-75% of cases) which may trigger compensatory EGFR activation when blocked, compounded by dense stromal barriers limiting drug penetration. The neutral effects in lung and colorectal cancers could reflect opposing β1/β2-AR signaling balances or unmeasured microbiome interactions that modulate adrenergic responses. These differential patterns underscore the importance of tumor-specific neuroimmune profiling to identify patients most likely to benefit from β-blocker therapy.

The bidirectional communication between the nervous and immune systems, termed cancer neuroimmunology, provides a framework for interpreting our results. Catecholamines promote tumor proliferation and angiogenesis via β-adrenergic receptors (β-ARs) (96). BBs inhibit these pathways, as seen in breast cancer models where propranolol reduced metastasis by downregulating MMPs and VEGF. Sympathetic overactivation suppresses antitumor immunity by impairing dendritic cell function and increasing regulatory T cells (Tregs). BBs reverse these effects, as demonstrated by enhanced CD8+ T-cell activity in preclinical studies. Tumors secrete neurotrophic factors NGF to recruit adrenergic nerves, creating a feed-forward loop (97). BBs may disrupt this by inhibiting nerve sprouting, as suggested by our analysis of NGF-driven innervation (HR = 1.15, 95% CI: 1.03–1.27). Chronic stress elevates catecholamines, which correlate with worse outcomes. BBs may mitigate this by reducing systemic stress responses, though patient-specific factors (e.g., genetic polymorphisms in β-ARs) could modulate efficacy (98).

### 4.3 Limitations

This meta-analysis has several limitations that warrant consideration. First, substantial heterogeneity (I² up to 88%) was observed, likely due to clinical diversity in cancer types, stages, and β-blocker regimens, as well as methodological differences in confounder adjustment across studies. Egger’s regression test (p = 0.03) confirmed small-study effects, particularly for survival outcomes (OS/CSS). Trim-and-fill analysis suggested 5 potentially “missing” negative studies, but the adjusted pooled HR (0.99, 95% CI: 0.94–1.04) remained non-significant. Potential immortal time bias in retrospective studies may have overestimated survival benefits (99). Second, funnel plot asymmetry suggested possible publication bias, with underrepresentation of small studies reporting null effects, while selective outcome reporting limited mechanistic insights as only 32% of studies included immune biomarker data. Funnel plot asymmetry and Egger’s tests suggest selective reporting of positive outcomes, particularly in smaller studies. While trim-and-fill adjustment attenuated pooled effects, residual bias may persist for understudied cancers. Third, the inability to establish temporal relationships due to lack of serial immune profiling in most studies constrained causal interpretations of β-blocker effects. Additionally, generalizability may be limited by the predominance of North American/European populations (87% of studies) and potential era effects as earlier studies might not reflect current immunotherapy practices (100).

## 5 Conclusion

This meta-analysis synthesizes a rapidly evolving body of evidence on BBs in cancer neuroimmunology. While no universal survival benefit was observed, significant subgroup effects—particularly in immunogenic tumors and immunotherapy recipients—support further investigation. The dual role of BBs as direct tumor suppressors and immune enhancers positions them as unique therapeutic agents, but their clinical translation requires precision medicine approaches (101). By addressing current limitations through rigorous RCTs and mechanistic studies, the oncology community can unlock the full potential of this paradigm-shifting strategy.

## Data Availability

The original contributions presented in the study are included in the article/**Supplementary Material**. Further inquiries can be directed to the corresponding authors.
