# Supplementary material for: Impact of beta blockers on cancer neuroimmunology: a systematic review and meta-analysis of survival outcomes and immune modulation
Source: Front Immunol. 2025 Aug 6;16:1635331. doi: 10.3389/fimmu.2025.1635331 (PMC12364651; doi:10.3389/fimmu.2025.1635331)
Supplement: Supplementary file 1 [file DataSheet1.docx]

Supplementary documents 1 Overall survival among cancer patients’ sensitivity analysis

Supplementary documents 1 Cancer-specific survival among cancer patients’ sensitivity analysis

Supplementary documents 3 Neurotrophic factors and tumor progression sensitivity analysis

Supplementary documents 4 Beta blockers and immune checkpoint inhibitors sensitivity analysis

Supplementary documents 5 Patients receiving combo therapy sensitivity analysis

(beta blockers + ICIs) vs. ICIs alone

Supplementary documents 6 PD-1/PD-L1 expression sensitivity analysis

Supplementary documents 7 β-adrenergic receptor expression sensitivity analysis
