# Supplementary material for: Impact of beta blockers on cancer neuroimmunology: a systematic review and meta-analysis of survival outcomes and immune modulation
Source: Front Immunol. 2025 Aug 6;16:1635331. doi: 10.3389/fimmu.2025.1635331 (PMC12364651; doi:10.3389/fimmu.2025.1635331)
Supplement: Supplementary file 2 [file Table1.docx]

Supplementary documents

Table 1 Summary of Key Meta-Analysis Findings

| **Outcome** | **No. Studies** | **Pooled HR/OR (95% CI)** | **I² (%)** |
| --- | --- | --- | --- |
| Overall Survival | 79 | 0.97 (0.92–1.02) | 80 |
|  |  |  |  |
| Cancer-Specific Survival | 40 | 0.95 (0.90–1.01) | 78 |
|  |  |  |  |
| BB + ICIs vs. ICIs Alone | 12 | 0.91 (0.85–0.98)* | 88 |
|  |  |  |  |
| PD-L1 Expression | 12 | OR=1.29 (1.10–1.52)* | 45 |
| CD8+ T-cell Infiltration | 9 | SMD=0.41 (0.22–0.60)* | 32 |
